# Supplementary material for: Asynchronous magnetic resonance elastography: Shear wave speed reconstruction using noise correlation of incoherent waves
Source: Magn Reson Med. 2022 Oct 27;89(3):990–1001. doi: 10.1002/mrm.29502 (PMC9792433; doi:10.1002/mrm.29502)
Supplement: Supplementary file 1 — DATA S1 MATLAB code used to generate the numerical simulations of Figure 1 [file MRM-89-990-s002.zip › k-Wave/helpfiles/makeBowl.html]

makeBowl :: Functions (k-Wave)


# makeBowl

Create a binary map of a bowl within a 3D grid.

## Syntax

```
bowl = makeBowl(grid_size, bowl_pos, radius, diameter, focus_pos)
bowl = makeBowl(grid_size, bowl_pos, radius, diameter, focus_pos, ...)
```

## Description

`makeBowl` creates a binary map of a bowl from a symmetric spherical shell within a three-dimensional grid. The bowl position is denoted by 1's in the matrix with 0's elsewhere. The bowl surface within the Cartesian grid is found using a bi-directional line search along each row and column to find the grid point with radius closest to the desired radius.

The position of the bowl is set by `bowl_pos`, which corresponds to the center of the rear bowl surface. The orientation of the bowl is set by `focus_pos`, which corresponds to any point on the axis of the bowl (note, this must not be equal to `bowl_pos`). It is assumed that the solid angle of the bowl is equal to or less than 2\*pi steradians. If the `radius` is set to `inf`, a disc is generated.

In some cases, the generated bowl will not be simply connected, and there will be a small number of overlapping grid points. To remove these points, set the optional input 'RemoveOverlap' to true.

## Examples

```
% define parameters
grid_size = [32, 64, 64];
bowl_pos  = [1, 32, 32];
radius    = 60;
diameter  = 61;
focus_pos = [32, 32, 32];

% create bowl
makeBowl(grid_size, bowl_pos, radius, diameter, focus_pos, 'Plot', true);
```

```
% define parameters
grid_size = [128, 128, 128];
bowl_pos  = [80, 80, 80];
radius    = 60;
diameter  = 81;
focus_pos = [1, 1, 1];

% create bowl
makeBowl(grid_size, bowl_pos, radius, diameter, focus_pos, 'Plot', true);
```

## Inputs

|  |  |
| --- | --- |
| `grid_size` | size of the 3D grid given as a three element vector [Nx, Ny, Nz] [grid points] |
| `bowl_pos` | centre of the rear surface of the bowl given as a three element vector [bx, by, bz] [grid points] |
| `radius` | radius of curvature of the bowl [grid points] |
| `diameter` | aperture diameter of the bowl [grid points] |
| `focus_pos` | any point on the beam axis of the bowl given as a three element vector [fx, fy, fz] [grid points] |

## Optional Inputs

Optional 'string', value pairs that may be used to modify the default computational settings.

| Input | Valid Settings | Default | Description |
| --- | --- | --- | --- |
| `'Binary'` | *(Boolean scalar)* | `false` | Boolean controlling whether the bowl map is returned as a double precision matrix (false) or a logical matrix (true). |
| `'Plot'` | *(Boolean scalar)* | `false` | Boolean controlling whether the bowl is plotted using `voxelPlot`. |
| `'RemoveOverlap'` | *(Boolean scalar)* | `false` | Boolean controlling whether overlapped grid points are removed. |

## Outputs

|  |  |
| --- | --- |
| `bowl` | 3D binary map of a bowl |

## See Also

`makeArc`, `makeBall`, `makeMultiBowl`, `makeSphere`, `makeSphericalSection`
